# Titanium dioxide nanotubes incorporated into bleaching agents: physicochemical characterization and enamel color change

**DOI:** 10.1590/1678-7757-2019-0771

**Published:** 2020-06-24

**Authors:** Naianne Ramos MONTEIRO, Rosanna Tarkany BASTING, Flávia Lucisano Botelho do AMARAL, Fabiana Mantovani Gomes FRANÇA, Cecilia Pedroso TURSSI, Orisson Ponce GOMES, Paulo Noronha LISBOA, Kamila Rosamilia KANTOVITZ, Roberta Tarkany BASTING

**Affiliations:** 1 Faculdade São Leopoldo Mandic CampinasSP Brasil Faculdade São Leopoldo Mandic, Campinas, SP, Brasil.; 2 Universidade Estadual Paulista Faculdade de Ciências BauruSP Brasil Universidade Estadual Paulista (UNESP) - Faculdade de Ciências, Bauru, SP, Brasil.

**Keywords:** Carbamide peroxide, Color, Hydrogen peroxide

## Abstract

Titanium dioxide nanotubes are nanostructures that can accelerate the oxidation reaction of bleaching procedures and promote a more effective whitening effect.

**Objective:**

This study evaluated physicochemical properties of bleaching agents incorporated with titanium dioxide (TiO_2_) nanotubes, and the effects on tooth color change at different periods.

**Methodology:**

40 premolars were treated according to the following groups (n=10): CP - 10% carbamide peroxide (1 hour daily/21 days); CPN - CP incorporated into TiO_2_; HP - 40% hydrogen peroxide (three 40-minute sessions/7 days apart); HPN - HP incorporated into TiO_2_. Color shade was evaluated at five different periods (baseline, after 7, 14 and 21 days of bleaching, and 7 days after end of treatment) according to Vita Classical, CIELab and CIEDE2000 scales. Mean particle size (P), polydispersity (PO) and zeta potential (ZP) were evaluated using dynamic light scattering. Data on the different variables were analyzed by mixed model tests for measures repeated in time (ZP e L*), generalized linear models for measures repeated in time (P, PO, Vita Classical and b*), and Friedman and Mann-Whitney tests (a* and color change/ΔE and ΔE_00_).

**Results:**

CP and CPN presented higher P, higher PO and lower ZP than HP and HPN (p≤0.05). All groups showed a significant decrease in Vita Classical color scores after 7 days of bleaching (p<0.05), and HPN presented a greater significant reduction than the other groups. L* increased in TiO_2_ presence, in all groups, without any differences (p>0.05) in bleaching time. A significant reduction occurred in the a* and b* values for all the groups, and HPN presented lower a* and b* values (p<0.05) than CPN. ΔE was clinically noticeable after 7 days, in all groups, and all groups resulted in a perceptible color change according to ΔE_00_.

**Conclusion:**

TiO_2_ did not influence physicochemical properties of the bleaching agents. HPN presented more effective tooth bleaching than CPN.

## Introduction

Dental sensitivity is one of the adverse effects directly related to whitening treatment^1-6^ and to concentration of the whitening agent, as well as time frame, application technique and composition of the whitening agent.^1-6^ High concentrations of hydrogen peroxide seem to increase the penetration and diffusion of peroxides and their by-products, leading to more intense inflammatory response and/or greater sensitivity.^1-6^

Some recommended methods to minimize sensitivity include using lower concentrations of bleaching agents,^6^ shortening the application time^7^ and introducing catalysts or accelerators of the oxidation reaction, such as sodium, potassium, iron, and manganese compounds, as well as titanium and graphene-based nanoparticles.^8-11^The use of catalysts or accelerators could provide a more pronounced bleaching effect in a shorter time, thus, achieving more efficient bleaching results, while requiring a lower hydrogen peroxide concentration.

Among the components used as catalysts for the oxidation reaction, titanium dioxide (TiO_2_) is biocompatible and it has antimicrobial properties,^12^ in addition to its known use as a pigment in cosmetics and food additives.^13,14^ When associated to a light source, it excites electrons and generates oxygen ions, producing superoxide.^15^ The catalytic action of TiO_2_ has strong oxidative power and high chemical stability, and being influenced by reactions with molecules on its surface. This process can be influenced by several factors, such as catalyst concentration, particle size and shape, pH, and surface area of the substrate.^15,16^

Because of the catalytic action of TiO_2_, some studies^17,18^ have found that its incorporation in in-office bleaching agents with lower concentrations of hydrogen peroxide (3.5%) and irradiation by light can promote a greater release of free radicals, and, consequently, more effective and clinically noticeable bleaching effect than non-incorporation of TiO_2_. However, it may be suggested that the incorporation of TiO_2_ in higher hydrogen peroxide concentrations than those used for the in-office bleaching technique could decrease the application time, and prevent against the common effect of dental sensitivity related to this procedure.

In most studies,^19-20^ TiO_2_ particles incorporated into bleaching agents have a spherical nanostructure, which tends to form clusters^12^that may compromise the effectiveness of the agent. However, TiO_2_ nanotubes, characterized by a hollow structure and a high relationship between surface and particle volume,^15,21^ could offer more benefits regarding the catalytic reaction promoted by the nanoparticle, even in the absence of light.^16^ This shape of the nanotube thus provides a larger surface area, and produces a greater number of radicals resulting from the increase in amount of active reaction sites for radical production. This suggests that the long one-dimensional structure of nanotubes is a better option.^15^

By adding a new component to the bleaching gel, like TiO_2_ nanotubes, the resulting physicochemical characterization may be altered. This could lead to sedimentation or aggregation of particles, which, in turn, could influence the expected effectiveness. Thus, this study aims to evaluate physicochemical characteristics of whitening agents containing 10% carbamide peroxide and 40% hydrogen peroxide when incorporated in TiO_2_ nanotubes, and the effects on the color change of the dental structure at different periods. The null hypotheses evaluated were that the addition of TiO_2_ nanotubes to whitening agents containing 10% carbamide peroxide or 40% hydrogen peroxide did not influence: 1) pH properties, particle size, polydispersibility or zeta potential of the whitening agents; 2) color alteration of the dental structure at different whitening periods.

## Methodology

### Selection of teeth, specimen preparation and distribution among groups

After approval of the study by the Research Ethics Committee, and in accordance with the World Medical Association Declaration of Helsinki (CAAE number 85659418.6.0000.5374), 65 healthy human premolars were selected and maintained frozen until use, but for no longer than 5 months. Firstly, periodontal ligament and debris were removed from the dental surface with periodontal curettes, and the teeth were evaluated using a stereoscopic magnifying glass (Eikonal Equip. Optical and Analytical, model EK3ST, São Paulo, SP, Brazil), magnified 20-fold in their entire length; those with cracks, wear and/or fractures were excluded.

Teeth were positioned in PVC molds (20 mm diameter and 20 mm high), the root portion was fixed to the amelocemental junction with self-curing acrylic resin (VipiFlash, Vipi, Pirassununga, SP, Brazil), and the long axis of the tooth was kept perpendicular to the horizontal plane. PVC molds were removed after resin polymerization.

A spectrophotometer (VITA Easyshade® Advance, Vita, Germany) was used to perform initial color evaluation applying the Vita Classical scale as criterion, after which the very light or very dark teeth were excluded. A total of 40 teeth were selected with colors that included A2, A3, B3, and A3.5, and they were distributed homogeneously among the four groups investigated (n=10): CP-10% carbamide peroxide; CPN-10% carbamide peroxide incorporated with TiO_2_ nanotubes; HP-40% hydrogen peroxide; and HPN-40% hydrogen peroxide incorporated with TiO_2_ nanotubes. The teeth were maintained at 37°C for 7 days in a bacteriological incubator before the start of the experiment, and then individually immersed in an artificial saliva solution (20 mL), composed of 1.5 mMol/L Ca; 50 mMol/L KCl; 0.9mMol/L PO_4_; 20 mMol/L Tris buffer (pH=7).^22^

### Specification of bleaching agents, obtaining of TiO2 nanotubes, and incorporation

**Figura 1:**
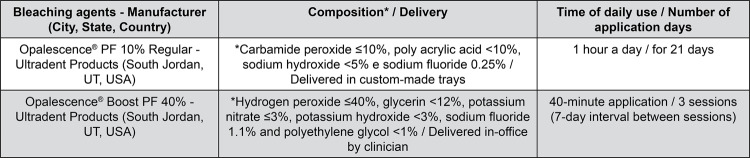
Bleaching agents, manufacturers, compositions, and delivery *According to the MSDS (Material Safety Data Sheet) of each product.

The bleaching agents used in this study, their compositions, time of daily use, and total treatment time, are described in Figure 1.

TiO_2_ nanotubes were formed from a single rolled sheet (~10 nm diameter and ~200 nm long), and they were produced in spiral structures; they were synthesized using the alkaline method.^23^ Nanotubes were prepared by mixing 12 g of TiO_2_ anatase phase, 99% purity, with 200 mL of 10 M NaOH. This mixture was maintained at 120°C for 24 h in an open Teflon container, placed in a glycerin bath, and heated with a mantle heater. The syntheses were carried out at ambient pressure, where only precursor reagents were subjected to alkaline treatment. After treatment, the mixture was washed repeatedly with 0.1 M hydrochloric acid and deionized water to remove the sodium ions. Next, the pH of the solution was adjusted to 7.^23^

TiO_2_ nanotubes were weighed on a 0.1 mg precision scale (BEL Engineering, Monza, Milan, Italy) and added manually to 9.9 mg of bleaching agents (CP or HP), at a concentration of 1%.^18,20^ The nanotubes were incorporated into the bleaching agents by spatulation (plastic spatula) on waterproof paper for 1 minute. Then, the resulting content was deposited into a sterile, disposable syringe. The TiO_2_ was incorporated into the bleaching agents right before each application.

### Physicochemical characterization of bleaching agents: pH analysis, average particle size, polydispersibility and zeta potential

The pH values were obtained in triplicate for the bleaching agents, according to respective measurement periods. The pH of the carbamide peroxide-based agents was measured initially after 30 minutes (half of the application time), and lastly after 1 hour (total application time). The pH of the hydrogen peroxide-based agents was measured initially after 20 minutes, and lastly after 40 minutes (total application time). The pH of both agents was measured by a table-top pH meter (MS Tecnopon Equipamentos Especiais, model: RbPH-210, Piracicaba, SP, Brazil). pH of the bleaching agents incorporated into TiO2 was evaluated immediately after manipulation of the prepared materials.

The average particle size of the whitening gels (hydrogen peroxide and carbamide peroxide) with or without the addition of 1% TiO_2_ nanotubes, polydispersibility (heterogeneity of the molecule) and zeta potential (colloidal stability) were evaluated by dynamic light scattering^24^ (Zetasizer Nano ZS, Malvern Instruments, Malvern, United Kingdom) in triplicate. No additional preparations of bleaching agents with or without TiO_2_ were necessary for these analyses. The zeta potential was evaluated to measure colloidal stability, using Helmholtz-Smoluchowski model,^25^ which measures the electrophoretic mobility of dispersed particles in the applied electric field.

The zeta potential analysis was performed by laser electrophoresis, with 30 runs per measurement at 25°C. The zeta potentials were estimated automatically using electrophoretic mobility, with the Smoluchowski approach: UE=2*ε*z*f(ka)/3*η→z≈UE*η/ε, where UE is the electrophoretic mobility, ε the dielectric constant, z is the zeta potential, f (ka) is the function of Henry, and η is the viscosity.^25^

The analyses (particle size, polydispersibility, and zeta potential) were performed in triplicate at 25°C and three times after the preparation of the bleaching agents with or without the addition of TiO_2_ nanotubes at 1%, namely: immediately after, 14 days, and 28 days.

### Bleaching protocol

The teeth were removed from the artificial saliva solution before each application of bleaching agent and dried with air jets for 5 seconds. Trays for each specimen of CP and CPN groups were made in a vacuum laminator (P7, Bio-Art Dental Equipment Ltd. São Carlos, Brazil), using a 1-mm-thick ethylene vinyl acetate (EVA) rubber plate (Soft, Bio-Art Dental Equipment Ltd). An aliquot of 0.03 ml of gel was placed in the middle third inside the tray, corresponding to the labial surface of the tooth. The tray was fitted over the tooth and remained in position for 1 hour.^8^ At the end of this time, the tray was removed, the tooth was washed with water for 10 seconds, and then returned to the artificial saliva solution, which was renewed every two days. The bleaching protocol was performed for 21 consecutive days.

Each tooth from HP and HPN groups received a 0.03 mL layer of bleaching agent on the vestibular surface. The agent remained on the dental surface for 40 minutes without replenishing the gel.^3,4^ At the end of the process, the gel was removed from the dental surface using a surgical aspiration tip, cleaned with gauze and rinsed with water for 10 seconds. The bleaching treatment was performed twice again, at 7-day intervals between sessions (total of three sessions of bleaching application). After completing the bleaching treatment, all teeth were stored in artificial saliva solution for 7 days, changing the solution every two days.

### Tooth Color Shade Evaluation

A spectrophotometer (VITA Easyshade^®^ Advance, Vita, Germany) was used to measure color at the middle third of the labial surface of the teeth. Tooth color was verified using the Vita Classical shade guide, and parameters L*, a*, and b* from the CIEL*a*b* system at different times, namely: T1-before bleaching procedure (baseline); T2-after 7 days of bleaching procedures; T3-after 14 days of bleaching; T4-after 21 days of bleaching; T5-7 days after the end of bleaching. The specimens were positioned inside a box with a white background for standardization of illumination, to perform the color measurements, conducted by the same rater at all times.

These measurements were duplicated at each period to ensure accuracy. When the two readings were the same for the Vita Classical scale, the value and other parameters (CIEL*a*b*) were recorded after the second reading. If the two readings did not match, a new measurement was carried out until agreement was achieved between readings.

The tooth color obtained from the Vita Classical scale was converted into numeric values, as previously established,^1,2^ according to an arrangement of colors from number 1 (shade B1) to 16 (shade C4),ordered by brightness or value: B1, A1, B2, D2, A2, C1, C2, D4, A3, D3, B3, A3.5, B4, C3, A4 and C4. Thus, the lower the numerical value, the higher the brightness, and the whiter the tooth.

After obtaining the values of ΔL*, Δa*, and Δb* for each group and period, ΔE was estimated using the following mathematical formula: ΔE = √((ΔL)^2^ + (Δa)^2^ +(Δb)^2^), where ΔE is the color change; ΔL*=L*_final_–L*_initial_; Δa*=a*final–a*_initial_; Δb*=b*_final_–b*_initial_. The value of ΔE>2.7 was considered as clinically noticeable.^26^Color change was also evaluated by CIEDE2000 (ΔE_00_), which uses h (hue) and C (chroma) values.^27^ ΔE_00_ values of 0.8 and 1.8 were adopted as 50:50% perceptibility and acceptability thresholds.^26^

### Statistical Analysis

The sample (n=3) for particle size, polydispersity and zeta potential data provided a power of 0.80 to test a 5% level of significance and an effect size higher than 1.0.^28^ The sample size for color evaluation (n=10) also provided a power of 0.80 to test for a mean size effect higher than 0.47 for color parameters (Vita Classical, L*, a* and b*) and 1.4 for color change (ΔE and ΔE_00_). The estimation were performed by G*Power software.^29^

Data on zeta potential were analyzed by mixed models for measures repeated in time, and they were presented as means and standard deviations. Generalized linear models for measures repeated in time were used, since particle size and polydispersity data did not meet the assumptions of parametric analysis, which were presented as medians, and minimum and maximum values.

Data on L* parameter were analyzed by mixed model tests for measures repeated in time. The color data from Vita Classical scale and b* parameter did not meet the presupposition for parametric analysis. They were analyzed by generalized linear models, considering the following factors: bleaching agent, presence of nanotubes and measures repeated in time. Since data for the a* parameter and color change (ΔE and ΔE_00_) did not meet the assumptions of a parametric analysis, Friedman tests were applied for time comparisons and the Mann-Whitney test for comparisons among bleaching agents and between groups with or without nanotubes.

All the analyses were performed with SAS^30^ and R^31^ software tools, at 5% significance level.

## Results

Bleaching agents presented pH value close to neutral, which remained similar and stable over the time of application, regardless of TiO_2_ incorporation (Table 1).

**Table 1:** Means of pH value according to group and time, of application

Bleaching agent	Time		
	Baseline	Half of application time *	End of application time**
CP	6.6	6.7	6.7
CPN	6.6	6.7	6.6
HP	7.0	7.2	7.3
HPN	7.3	7.4	7.3

* CP and CPN groups: 30 minutes; HP and HPN groups: 20 minutes.

** CP and CPN groups: 1 hour; HP and HPN groups: 40 minutes.

The mean particle size showed stability in CPN group during the evaluation time (Table 2). HPN showed a decrease in mean particle size over a 14-day period, and a subsequent increase at 28 days (p=0.0037), without a significant difference from the initial time. The mean particle size was significantly larger in the CP group (p=0.0007), regardless of period and presence or absence of TiO_2_. As for polydispersibility (Table 2), CP and CPN showed a significant decrease (p=0.0138) over time, whereas the values remained statistically similar for HP and HPN. No significant difference was observed between CP and CPN groups, or between HP and HPN groups (p=0.1286). Greater polydispersibility was found in the CP and CPN groups, regardless of time, compared with the HP and HPN groups (p=0.0012). Zeta potential (Table 2) of CP, HP and HPN did not vary significantly over time, whereas a significant decrease occurred in CPN (p<0.0001). Zeta potential was significantly lower in the CP and CPN groups, compared with the HP and HPN groups (p=0.0012), regardless of time.

**Table 2:** Median (minimum value; maximum value) particle size and polydispersibility, and mean (standard deviation) zeta potential, as a function of the bleaching agent, addition of TiO2 and time-point

	Bleaching agent	Time		
		Baseline	14 days	28 days

Average particle size (in nm)	CP	3274.00 (2066.00; 3480.00)^Aa^	1587.00 (1385.00; 1739,00)^Ba^	662.60 (644.50; 810.70)Cb
	CPN	1238.00 (1092.00; 1281.00)^Ab^	1221.00 (1128.00; 1814.00)^Aa^	1123.00 (1031.00; 1460.00)^Aa^
	HP	*651.10 (584.70; 738.70)^Aa^	*730.60 (708.20; 732.70)^Aa^	*619.30 (563.30; 623.40)^Ba^
	HPN	*634.50 (545.50; 655.00)^Aa^	*475.80 (438.50; 494.20)^Bb^	*565.70 (562.90; 567.30)^Ab^
	p(bleaching agent)=0.0007; p(TiO_2_)=0.0043; p(bleaching agent x TiO_2_)=0.6655; p(time)=0.0037; p(bleaching agent x time)=0.0039; p(TiO_2_ x time)=0.0047; p(bleaching agent x TiO_2_ x time)=0.0055.			

Poly-dispersity index	CP	1.00 (1.00; 1.00)^Aa^	0.79 (0.77; 0.79)^Ba^	0.69 (0.63; 0.81)^Ca^
	CPN	1.00 (0.83; 1.00)A^Aa^	0.79 (0.70; 1.00)^Ba^	0.66 (0.66; 0.68)^Ca^
	HP	*0.46 (0.45; 0.52)^Aa^	*0.30 (0.09; 0.53)^Aa^	*0.48 (0.44; 0.69)^Aa^
	HPN	*0.54 (0.50; 0.59)^Aa^	*0.50 (0.40; 0.61)^Aa^	*0.52 (0.49; 0.62)^Aa^

	p(bleaching agent)=0.0012; p(TiO_2_)=0.2033; p(bleaching agent x TiO_2_)=0.1286; p(time)=0.0138; p(bleaching agent x time)=0.0243; p(TiO_2_ x time)=0.3270; p(bleaching agent x TiO_2_ x time)=0.6617.			

Zeta potential (in Mv)	CP	-47.40 (1.35)^Aa^	-50.87 (5.13)^Aa^	-53.77 (1.20)^Aa^
	CPN	-43.17 (0.83)^Aa^	-56.63 (6.33)^Ba^	-68.73 (4.53)^Cb^
	HP	*-30.43 (0.70)^Aa^	*-36.30 (0.56)^Aa^	*-30.43 (2.11)^Aa^
	HPN	*-35.00 (2.31)^Aa^	*-34.93 (0.15)^Aa^	*-36.13 (1.31)^Aa^

	p(bleaching agent)<0.0001; p(TiO_2_)=0.0012; p(bleaching agent x TiO_2_)=0.1795; p(time)<0.0001; p(bleaching agent x time)<0.0001; p(TiO_2_ x time)=0.0023; p(bleaching agent x TiO_2_ x time)=0.0047.			


Groups with medians or means followed by distinct letters (uppercase horizontally and lowercase vertically, comparing groups with and without nanotubes for each bleaching agent) differ from each other (p≤0.05). * Values differ from CP, in reference to nanotubes and time-points (p≤0.05).

The Vita Classical scale color score decreased significantly for all groups after 7 days, but there were no differences between 7 and 14 days (Table 3). After 21 days, a significant decrease occurred in the score related to the previous period (14 days) only for CP. At 7 days, after the end of whitening, only CP and HP showed a significant decrease in the color score, in relation to the 7-day whitening period. At the period of 21 days of whitening and 7 days after whitening, CPN presented a higher color score than CP. At 7 days of whitening, HPN presented a lower color score than HP. At the periods of 7, 14, 21 days and after 7 days of whitening, HPN presented a score significantly lower than CPN. There was an increase in L* (Table 3) in all groups, especially after 7 days of evaluation, and a significant increase especially in CP and HP after 14 days. However, no significant differences occurred among the groups at any evaluation period.

**Table 3:** Mean (standard deviation) of Vita Classical color variables L* and b* as a function of group and of time-point, and median (minimum value; maximum value) of parameter a* as a function of group and time-point

	Bleaching agent		Time			
		Baseline	After 7 days	After 14 days	After 21 days	7 days after end of bleaching treatment

Vita Classical	CP	10.40 (2.06)^A^	5.90 (3.28)^B^	6.10 (3.54)^B^	4.40 (2.63)^C^	4.40 (2.63)^C^
	CPN	10.50 (2.12)^A^	8.00 (4.00)^B^	8.00 (4.00)^B^	*7.90 (3.72)^B^	*8.20 (4.10)^B^
	HP	10.20 (2.10)^A^	6.40 (3.75)^B^	4.70 (3.59)^BC^	3.50 (2.80)^C^	4.30 (3.27)^C^
	HPN	9.90 (2.02)^A^	*$3.60 (2.06)^B^	$3.60 (2.22)^B^	$3.40 (2.72)^B^	$2.70 (0.95)^B^

L*	CP	80.95 (2.64)^C^	82.67 (3.74)^B^	85.82 (3.37)^A^	87.10 (3.54)^A^	84.52 (3.42)^AB^
	CPN	80.94 (2.04)^B^	84.33 (2.25)A	84.75 (4.24)^A^	85.81 (3.31)^A^	84.87 (2.69)^A^
	HP	81.17 (3.37)^C^	83.71 (2.81)^B^	87.09 (3.23)^A^	86.05 (2.44)^A^	85.33 (3.29)^AB^
	HPN	84.38 (1.70)^B^	86.11 (1.42)^A^	87.42 (2.48)^A^	87.08 (2.44)^A^	86.41 (2.47)^A^

a*	CP	1.20 (-0.60; 3.30)^A^	-0.60 (-2.30; 1.40)^AB^	-0.65 (-2.40; 1.10)^AB^	-0.80 (-2.60; 0.20)^B^	-0.70 (-2.30; 0.20)^B^
	CPN	1.30 (-1.20; 3.40)^A^	-0.10 (-2.40; 0.90)^B^	-0.35 (-2.20; 1.90)^AB^	-0.15 (-2.10; 1.80)^AB^	-0.40 -2.10; 1.60)^B^
	HP	0.40 (-1.60; 2.70)^A^	-1.00 (-2.30; 1.80)^AB^	-2.05 (-3.10; 0.60)^B^	-2.00 (-3.10; 1.00)^B^	-1.55 (-2.50; 0.30)^B^
	HPN	0.45 (-1.50; 1.70)^A^	$-1.25 (-2.70; -0.40)^B^	$-1.25 (-2.70; -0.20)^B^	$-1.45 (-3.10; 0.10)^B^	$-1.40 (-2.90; -0.80)^B^

b*	CP	27.00 (4.18)^A^	21.93 (3.12)^B^	22.76 (3.65)^B^	21.75 (2.58)^B^	21.70 (4.18)^B^
	CPN	27.75 (4.91)^A^	25.52 (6.11)^AB^	25.07 (5.17)^B^	*26.40 (6.06)^A^	24.64 (6.08)^B^
	HP	26.78 (5.83)^A^	21.81 (5.18)^B^	20.14 (3.76)^B^	19.78 (4.44)^B^	20.40 (4.22)^B^
	HPN	27.32 (5.36)^A^	20.37 (2.48)^BC^	$20.86 (3.12)^B^	$19.36 (3.24)^CD^	$18.63 (2.35)^D^

Means followed by different horizontal letters differ from one another (p<0.05).

*CPN mean values differ from CP, in reference to nanotubes and time-points (p≤0.05).

$HPN mean values differ from the CPN at the same time-point (p<0.05).

Over time, all groups presented a significant decrease in mean/median values for a* and b*. There were no significant differences among groups at the evaluation periods, except CPN, that presented a significantly higher b* value than CP, in 21 days of whitening. HPN had a significantly lower b* value than CPN at 14, 21 days and after 7 days of whitening. The CPN group had a b* value similar to the baseline at 21 days of whitening, whereas HPN group had a b* value significantly lower than that of the baseline at 14-day period, and similar to that obtained 7 days after whitening.

As for ΔE and ΔE_00_ (Table 4), they were significantly higher (p<0.05) in HPN than CPN at 7 days after whitening in relation to the baseline time, but ΔE was not significantly different among the groups at the other periods. Meanwhile, ΔE_00_ was significantly higher in HP than HPN only at 14 days of bleaching treatment (p<0.05).

**Table 4:** Median (minimum value; maximum value) color variation (CIEL*a*b* system and CIEDE2000) as a function of group and time period

	Bleaching agent	Time period			
		After 7 days – baseline	After 14 days – baseline	After 21 days – baseline	7 days after end of bleaching treatment – baseline

ΔE	CP	6.14 (1.97; 9.28)	7.26 (2.19; 13.25)	9.32 (3.71; 13.35)	8.09 (4.26; 11.88)
	CPN	5.34 (2.07; 9.61)	5.82 (1.73; 13.02)	5.32 (2.44; 13.07)	5.36 (2.43; 14.29)
	HP	7.01 (2.47; 12.16)	10.28 (5.00; 16.73)	9.61 (3.61; 18.13)	8.88 (6.26; 18.01)
	HPN	7.08 (2.17; 13.53)	7.35 (4.69; 14.84)	8.62 (4.01; 15.65)	$9.12 (4.37; 18.35)

ΔE00	CP	3.62 (1.85; 5.68)	4.23 (1.27; 8.07)	5.14 (2.25; 8.39)	5.32 (2.24; 7.53)
	CPN	2.87 (1.44; 4.70)	3.17 (1.12; 7.90)	3.33 (1.79; 7.76)	4.07 (1.61; 8.14)
	HP	4.28 (1.74; 7.18)	5.94 (3.34; 9.17)	5.54 (2.54; 9.81)	4.85 (3.51; 10.02)
	HPN	4.05 (1.53-5.69)	*4.16 (3.12-6.64)	4.86 (2.77; 6.98)	$5.50 (3.08; 8.07)

$HPN differs from the CPN at the same time-point (p<0.05).

*HPN differs from the HP at the same time-point (p<0.05).

## Discussion

When TiO_2_ nanotubes were incorporated into the bleaching agents, the pH was stable during the application time, and the gel pH remained close to neutral. Such as gel composition and temperature influence hydrogen peroxide decomposition,^32,33^ pH value can also affect the bleaching efficacy.^30,32^ It is well-known that the bleaching efficacy of hydrogen peroxide is directly proportional to pH, and that pH increases as the speed of the reaction to hydrogen peroxide decomposition increases, triggering the release of free radicals.^34^ The higher the pH, the greater the dissociation of hydrogen peroxide, leading to greater formation of reactive free radicals that improve bleaching efficacy.^35^ On the other hand, a more acidic pH promotes the stability of hydrogen peroxide, keeping it from decomposing, and favoring bleaching agent longevity during storage.^34^ Although it has been observed that the lower the pH of the bleaching agent, the greater the diffusion of peroxides in the dental structure, there is also a greater risk and intensity of dental sensitivity, compared with a whitening gel of more alkaline pH.^35,36^

The incorporation of TiO_2_ at a concentration of 1% also produced a gel with clinical handling characteristics, regarding viscosity, similar to those of the original commercial product. The properties of polydispersity and colloidal stability (zeta potential) were similar between gels. This indicates that there was no tendency of particles agglomeration in the whitening gel—leading to greater material stability—regardless of TiO_2_ presence. Despite the lack of agglomeration, CPN showed smaller particle size than CP at the initial period, leading us to reject the first null hypothesis of this study. The smaller particle size for carbamide peroxide incorporated into TiO_2_ nanotubes could be related to the chemical changes of the material, a variable that should be investigated in future studies.

Notably, different compositions of the bleaching agents in the HP and CP groups provide significantly different physical characteristics to the agents, as could be noticed at the initial period, in which the carbamide peroxide-based agents had larger particle sizes and polydispersibility, and lower zeta potential than the hydrogen peroxide-based agents. However, the addition of TiO_2_ to carbamide peroxide led to an agent with smaller mean particle size, although it did not influence the product’s polydispersibility characteristic.

The polydispersibility index is a parameter that defines the distribution of particles in the material, where greater uniformity of distribution is evidenced by lower values of polydispersion.^37,38^ The polydispersibility values for HP and HPN agents were significantly lower at all evaluation periods than those for CP and CPN agents, thus contributing to the lower probability of formation of agglomerates in the bleaching gel. In this sense, values of zeta potential above (+/-) 30 mV indicate stable molecules in suspension, since the surface load prevents the aggregation of particles.^39^ In this study, the zeta potential values showed good colloidal stability for all agents, with higher values for agents containing CP, considering that it is desirable for a system to present high surface load by generating repulsive forces that tend to prevent the aggregation of particles.^38^

Note that mean particle size, polydispersibility and zeta potential evaluations of HP and HPN agents were performed a second time after those done initially (14 and 28 days); leftover manipulated gel was stored for the same period of time and reevaluated, but it was not used. This procedure was different from the protocol recommended by the manufacturer and performed in this study methodology, in which the components of the two bleaching agent syringes should be manipulated only at time of use. Considering the limitations of this procedure, it is suggested that future studies investigate the effects of previous incorporation of TiO_2_ nanotubes in only one of the syringes, to support clinical process of handling the bleaching agent.

Analysis of whitening efficacy using Vita Classical scale showed that all treatments were effective considering their ability to promote tooth whitening, with a significant decrease in scores after 7 days of treatment. However, HPN group showed a greater reduction in Vita Classical scale scores (approximately 6 scores) soon after the first application of the bleaching agent, and maintained this result during the entire whitening treatment. The incorporation of TiO_2_ nanotubes may have contributed to the greater formation and release of reactive radicals early in the treatment, thus highlighting their catalytic action.^40,41^ Suyama, et al.^18^ (2009) also indicated that formulations that included hydrogen peroxide at 3.5% and the addition of TiO_2_ at concentrations lower than 0.1%, activated by visible light or ultraviolet light, were significantly more effective than only hydrogen peroxide at 3.5%.

TiO_2_ in its crystalline form can be found in three different phases, namely: anatase, rutile, and brookite. The anatase phase can be transformed into nanotubes, which is relevant as a catalytic agent and photocatalyst.^15,23,41^ The anatase phase of TiO_2_ nanoparticles is stable;^23^ this may also have contributed to pH properties, mean particle size, polydispersibility and zeta potential. Although no light source was used for photocatalysis of the agent, TiO_2_ particles at nanoscale may be reactive, even in the absence of activation sources,^16^ especially when associated to 40% hydrogen peroxide.^20^ The catalytic action of TiO_2_ has strong oxidative power and high chemical stability, and it is influenced by reactions with molecules on its surface.^15^ However, TiO_2_ nanotubes shape provides larger surface area, increasing the amount of superoxide radicals produced, such as O_2_- and hydroxyl radicals.^15^ This is expected to improve color change when associated to hydrogen peroxide agents. This finding was also identified in the study by Sakai, et al.^17^ (2007), who showed that gel with hydrogen peroxide at 3.5% and TiO_2_ (less than 1%), even without diode laser activation (405 nm), generated hydroxyl radicals, albeit in significantly smaller amounts than the irradiated group.

On the other hand, association of TiO_2_ to carbamide peroxide did not seem to show any potentiation of the bleaching effect when evaluating Vita Classical scale, since the reduction in the color score after 7 days was only 2 shades, after which the color remained stable throughout the treatment; moreover, this association proved less effectiveness than 10% carbamide peroxide at the end of the bleaching treatment. Lee, et al.^40^ (2005) suggested that hydrogen peroxide concentration affects the generation of reactive oxygen species when associated to TiO_2_, because lower TiO_2_concentrations in the bleaching agent do not seem to favor greater release of radicals that could enhance the bleaching effect. In this respect, Cuppini, et al.^20^ (2019) also identified greater effectiveness of TiO_2_ nanoparticles associated to a 35% versus 3.5% hydrogen peroxide concentration, showing that the improvement in bleaching efficacy may be associated to the hydrogen peroxide concentration and the amount of radicals generated with the incorporation of TiO_2_ nanotubes. Nevertheless, the addition of TiO_2_ at 0.1% in the same study^20^ was able to improve the performance of the bleaching gel, regardless of the hydrogen peroxide concentration.

Regarding the evaluation of color change according to CIEL*a*b* system, an increase in luminosity (L*) was observed in all groups, especially after 7 days. Cardoso, et al.^7^ (2010) showed that patients with a bleaching treatment using 10% carbamide peroxide with daily applications of one-hour were as satisfied as those who used it for eight hours a day.

As for coordinate a*, a decrease occurred in the median values of pigments in the red axis range in all groups not influenced by TiO_2_, since this parameter does not seem to be the most relevant factor when evaluating the bleaching effectiveness of a technique, as compared to parameter b*.^2^ Significantly lower b* values (less yellow teeth) were obtained for HPN compared to CPN (after 14 and 21 days of whitening, and 7 days after end of treatment), indicating that HPN performed better, possibly due to the higher concentration of hydrogen peroxide in the whitening agent.^20,41^

The color change (ΔE) values obtained by all the techniques were higher than 2.7 (minimum value of ΔE that is clinically perceptible), compared to the baseline, denoting the effectiveness of all bleaching procedures.^3,7^ However, the color change obtained by the CIEDE2000 formula (ΔE_00_) is better correlated with visual perception than the CIELAB.^26^ In this study, ΔE_00_ values between baseline and 7 days after end of the bleaching treatment (above 4.07) were higher than the 50:50% perceptibility threshold,^26^ showing their effectiveness in dental bleaching. By incorporating TiO_2_ nanotubes (10 mg) into the hydrogen peroxide at 3%, followed by irradiation with LED, Komatsu, et al.^15^ (2014) observed significantly higher color change values in the groups that had TiO_2_ nanotubes as a catalyst agent, compared with the groups with TiO_2_ in spherical form, or with the group without catalyst agent, which was not researched in this study. However, when Martín, et al.^42^ (2015) compared the whitening efficacy of one agent containing 35% hydrogen peroxide with another containing 6% hydrogen peroxide + TiO_2_ nanotubes doped with nitrogen (irradiated with hybrid LED/Laser light), they observed a similarity between the groups in the subjective analysis. Regarding the objective analysis, a remarkable difference of more than 2 ΔE units was observed, with higher values for the gel of higher concentration. In our study, the presence of TiO_2_ in the hydrogen peroxide agent promoted more significant color change than that of the CPN group, thus leading to the rejection of the second null hypothesis, since the addition of TiO_2_ nanotubes to the whitening agents influenced the color change of the dental structure at different whitening time-points. This effect may depend on the concentration of nanotubes, and whitening agent. The performance of TiO_2_ nanotubes may have been enhanced by the higher hydrogen peroxide concentration (40%).

The results of this study suggest that the inclusion of TiO_2_ nanotubes seems to be promising (especially in in-office bleaching), although the ideal relationship between TiO_2_ nanotubes and bleaching agent concentrations should be further investigated to provide more relevant improvement in the bleaching effect, and promote a safer and more effective procedure for patients, with fewer adverse effects. Furthermore, transmission electron microscopy analysis should be performed to determine the possibility of nanotubes agglomerating with other components of the bleaching agents, as well as further studies regarding the use of light to improve the bleaching efficacy with carbamide and hydrogen peroxides containing TiO_2_ nanotubes.

## Conclusion

The concentration of TiO_2_ nanotubes used in this study (1%) did not change the physicochemical properties of the hydrogen peroxide, which may have supported the greater color change observed for CPN.
